# *MAF2* Is Regulated by Temperature-Dependent Splicing and Represses Flowering at Low Temperatures in Parallel with *FLM*

**DOI:** 10.1371/journal.pone.0126516

**Published:** 2015-05-08

**Authors:** Chiara A. Airoldi, Mary McKay, Brendan Davies

**Affiliations:** Centre for Plant Sciences, University of Leeds, Leeds, United Kingdom; Ecole Normale Superieure, FRANCE

## Abstract

Plants enter their reproductive phase when the environmental conditions are favourable for the successful production of progeny. The transition from vegetative to reproductive phase is influenced by several environmental factors including ambient temperature. In the model plant *Arabidopsis thaliana*, *SHORT VEGETATIVE PHASE (SVP)* is critical for this pathway; *svp* mutants cannot modify their flowering time in response to ambient temperature. *SVP* encodes a MADS-box transcription factor that directly represses genes that promote flowering. SVP binds DNA in complexes with other MADS-box transcription factors, including FLOWERING LOCUS M (FLM), which acts with SVP to repress the floral transition at low temperatures. Small temperature changes post-transcriptionally regulate *FLM* through temperature-dependent alternative splicing (TD-AS). As ambient temperature increases, the predominant *FLM* splice isoform shifts to encode a protein incapable of exerting a repressive effect on flowering. Here we characterize a closely related MADS-box transcription factor, MADS AFFECTING FLOWERING2 (MAF2), which has independently evolved TD-AS. At low temperatures the most abundant *MAF2* splice variant encodes a protein that interacts with SVP to repress flowering. At increased temperature the relative abundance of splice isoforms shifts in favour of an intron-retaining variant that introduces a premature termination codon. We show that this isoform encodes a protein that cannot interact with SVP or repress flowering. At lower temperatures MAF2 and SVP repress flowering in parallel with FLM and SVP, providing an additional input to sense ambient temperature for the control of flowering.

## Introduction

The timing of the vegetative to reproductive phase transition in plants is influenced by many environmental stimuli. Perception and integration of a range of environmental signals maximises reproductive success and species fitness. Initiation of the reproductive phase is regulated by environmental variables, such as day-length and temperature, in addition to endogenous signals such as plant age [1] [2]. In *Arabidopsis thaliana* these diverse inputs are integrated by regulating the expression of a limited set of genes including *FLOWERING LOCUS T* (*FT*) and *SUPPRESSOR OF OVEREXPRESSION OF CONSTANS* (*SOC1*). *FT* and *SOC1* are therefore known as floral pathway integrators [3], because several pathways converge on these activators of flowering to translate endogenous and exogenous signals into the decision to flower.

Amongst these stimuli, temperature influences flowering time in two distinct ways. Exposure to prolonged periods of cold promotes flowering through a process known as vernalization [4]. Vernalization enables plants to identify the spring by distinguishing a long period of winter from a transient cold spell in autumn. Two related MADS-box transcription factors, FLOWERING LOCUS C (FLC) and MAF2 have been shown to act in complementary ways in the vernalization pathway to delay flowering until the plant has experienced extended periods of cold. FLC is a repressor of flowering and the amount of *FLC* expression varies in *Arabidopsis* ecotypes, determining their vernalization requirements [5]. Expression of *FLC* is increased in ecotypes with an active *FRIDGIDA* (*FRI*) gene, because FRI activates *FLC* as part of a transcription complex that binds to the *FLC* promoter [6] and by binding to mRNA cap binding proteins to link transcriptional regulation with RNA processing [7]. *FLC* expression is high in *FRI*+ ecotypes, which require vernalization to flower. In contrast, in the Columbia (*Col*) background, where *FRI* is mutated, *FLC* expression is low and vernalization is not necessary [8]. Like *FLC*, expression of *MAF2* is down-regulated after exposure to long periods of cold, although the differing expression kinetics mean that *MAF2* ensures that flowering remains repressed despite plants experiencing transient periods of cold sufficient to down-regulate *FLC*. *FLC* expression is abolished in *Col* after ten days of cold treatment, whereas abolition of *MAF2* expression requires 85 days of cold exposure [9]. Therefore one role of *MAF2* could be to prevent flowering from being induced by a short cold spell, sufficient to down-regulate *FLC* expression. FLC interacts with SVP and directly represses the floral pathway integrator genes *FT* and *SOC1*[10][11]. MAF2 is also capable of interacting with SVP and *maf2* mutants have elevated *SOC1* and *FT* expression [9][12].

However, temperature affects flowering time in another way, involving some of the same factors. Changes in the ambient temperature influence flowering time, repressing the floral transition at low temperatures and inducing it at high temperatures [13]. In *Arabidopsis* even small changes in ambient temperature can modify flowering time [14]. Recently, several genes have been shown to be involved in ambient temperature sensing in flowering; *FLOWERING TIME CONTROL LOCUS A* (*FCA*), *FVE*, *SVP*, *FLM* and *PHYTOCHROME INTERACTING FACTOR 4* (*PIF4*) [13][2].

*FVE* and *FCA* were among the first genes characterized as belonging to the thermosensory pathway [15]. FVE is part of a chromatin remodelling complex [16]. FCA is involved in cleavage and polyadenylation of mRNAs [17][18][19] and also promotes the processing of specific miRNAs, including miR172, which plays a role in temperature-regulated flowering [20]. miR172 inhibits the expression of several *AP2*-family transcription factors (e.g. *SCHLAFMÜTZE* (*SMZ*) [21], *TARGET OF EAT 1* (*TOE1*) and *TOE2* [22]), which would otherwise repress the floral pathway integrator, *FT*, and inhibit floral transition [23]. Since the abundance of miR172 is greater at 23°C than at 16°C [24], flowering is enhanced at the higher temperature by a reduction in the levels of the AP2 floral repressors leading to an increase in *FT*. The increase in miR172 abundance at 23°C is dependent on *FCA*, which is itself regulated by ambient temperature through both gene expression and protein stability [20].

In short days the phytochrome-interacting bHLH transcription factor, PIF4, induces flowering in response to elevated temperature, again by increasing *FT* expression [25]. Although both *PIF4* transcript levels and PIF4 protein stability are somewhat increased at higher temperatures, the enhanced ability of PIF4 to bind to and activate *FT* at higher temperatures is likely to be a more significant factor in its contribution to thermosensitivity [25]. The temperature dependency of PIF4 binding to *FT* is mediated at the chromatin level. Increasing temperature is associated with decreased H2A.Z-nucleosomes at the *FT* promoter [26], providing greater chromatin accessibility and binding of PIF4 [25].

Finally, multimeric complexes containing the MADS-box transcription factor SVP also play an important role in ambient temperature sensing, by repressing *FT* and *SOC1* expression at low temperatures, thereby delaying flowering [12][27][28]. With respect to flowering time, *svp* mutants are insensitive to ambient temperature from 16°C to 23°C [29]. *SVP*-mediated repression of flowering is alleviated at increased temperature, at least in part, because the SVP protein is degraded by the proteasome under these conditions [28]. SVP interacts with several MADS-box transcription factors of the FLC sub-family; FLC, MAF2, MAF4 and FLM [28][12][27], to repress the expression of *FT* and *SOC1* [12]. Strikingly, two of these interaction partners, *MAF2* and *FLM*, have been shown to use temperature-dependent alternative splicing (TD-AS) to transduce the ambient temperature signal into a regulatory effect on flowering time [14][30][28][27].

FLM independently represses flowering in response to both low ambient temperature and short days. The *flm* mutant is slightly early flowering in long days, but this effect is enhanced in short days, where *flm* flowers much earlier than wild type controls [31]. Furthermore, analysis of flowering time in *flm* mutants has shown that FLM represses flowering at low temperatures, but not at higher temperatures around 27°C [27][14]. In *Col FLM* produces two splice variants, *FLM-β* and *FLM-δ*, which generate proteins with differing abilities to repress the floral pathway integrator genes [27][28]. FLM-β can interact with SVP and repress *SOC1* whereas FLM-δ interacts with both SVP and FLM-β, but does not repress *SOC1*, acting instead to decrease the availability of repressive SVP and FLM-β. At 16°C the *FLM-β* isoform predominates, producing an active repressor of flowering. Increasing the temperature to 27°C causes the FLM-δ isoform to predominate, relieving the repression and allowing flowering.

*MAF2*, like *FLM*, is a major determinate of natural variation in *Arabidopsis* flowering time [32] which is also subjected to TD-AS to respond to ambient temperature and repress flowering at low temperatures [9] [30]. Here we further characterise the role of MAF2 in regulating the floral transition as part of an SVP-containing complex. Although *MAF2* and *FLM* have independently acquired the ability to sense temperature by TD-AS, we show that the effect of the *flm maf2* double mutant on thermosensitivity is similar to that of the *svp* single mutant, demonstrating that MAF2 and FLM both act with SVP to control the ambient temperature pathway.

## Results

### MAF2 requires SVP to repress flowering

**Fig 1:**
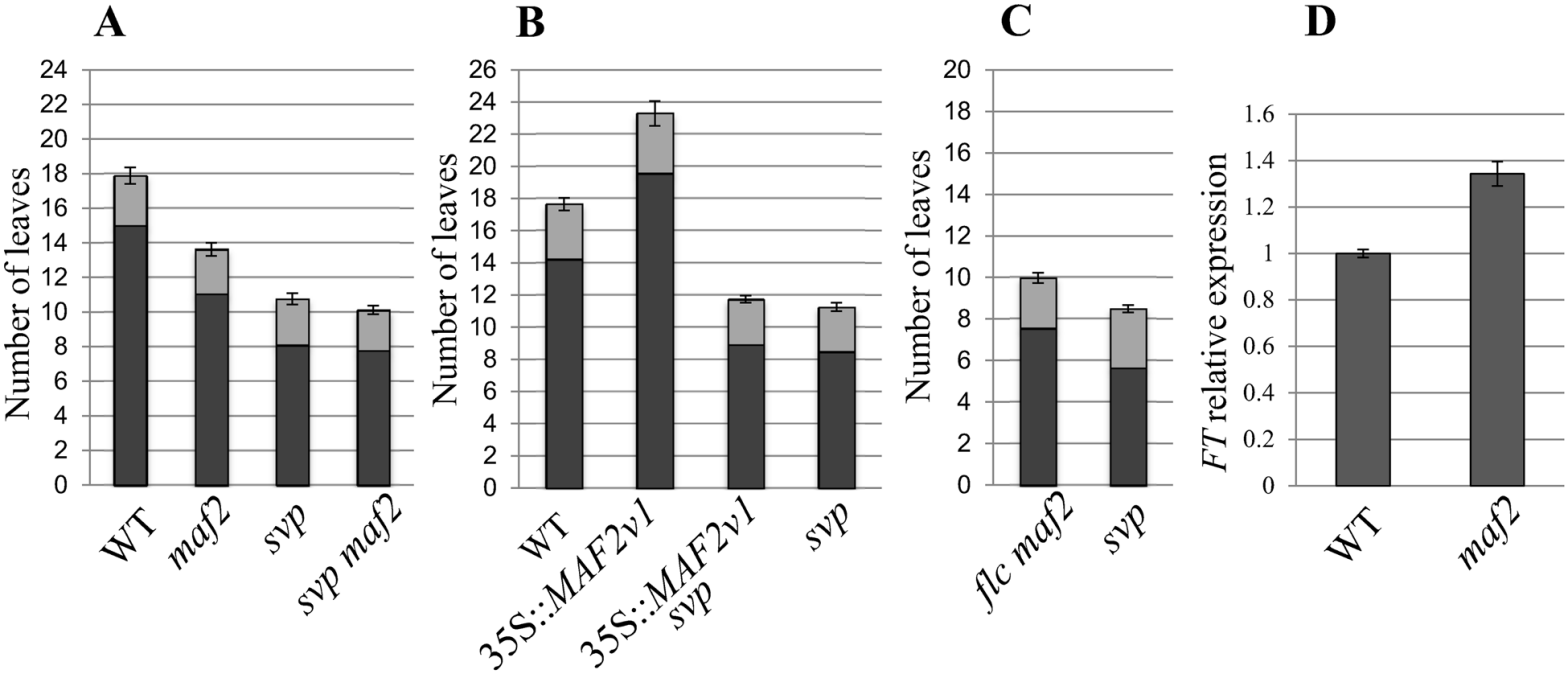
Comparison of flowering time in WT and mutant backgrounds. A,B,C, the columns indicate the number of rosette leaves (in black) plus the number of cauline leaves (in grey). Plants were grown at 21°C. A, Comparison of flowering time between WT and *maf2*, *maf2 svp* and *svp* mutants. (no. of plants analyzed 161) B, Flowering time of WT and plants overexpressing *MAF2var1* in WT and *svp* backgrounds (no. of plants analyzed 100, four independent transformants for each transgenic line). C, Flowering time of *flc maf2* double mutants compared to *svp* mutants (no. of plants analyzed 92). D, Quantitative RT-PCR of *FT* in WT and *maf2* backgrounds. Error bars represent the standard error.

The FLC and FLM proteins both interact with SVP to repress flowering [11][27]. Since MAF2 is also capable of interacting with SVP [12], we investigated the significance of SVP for MAF2 function by analysing flowering time in single and double mutants. Comparisons of flowering time in WT, *maf2*, *svp*, and *svp maf2* backgrounds revealed that the early flowering phenotype seen in *svp* is not significantly enhanced in the *svp maf2* double mutant (Fig 1A), suggesting that *MAF2* and *SVP* are in the same pathway and that the MAF2-SVP interaction is required for the repression of flowering by MAF2. Expression analyses show that *SVP* remains expressed in a *maf2* background, possibly even at a higher level than in wild type, and *MAF2* also remains expressed in an *svp* mutant background (S1 Fig). These results show that *MAF2* and *SVP* do not depend on each other for gene expression.

**Fig 2:**
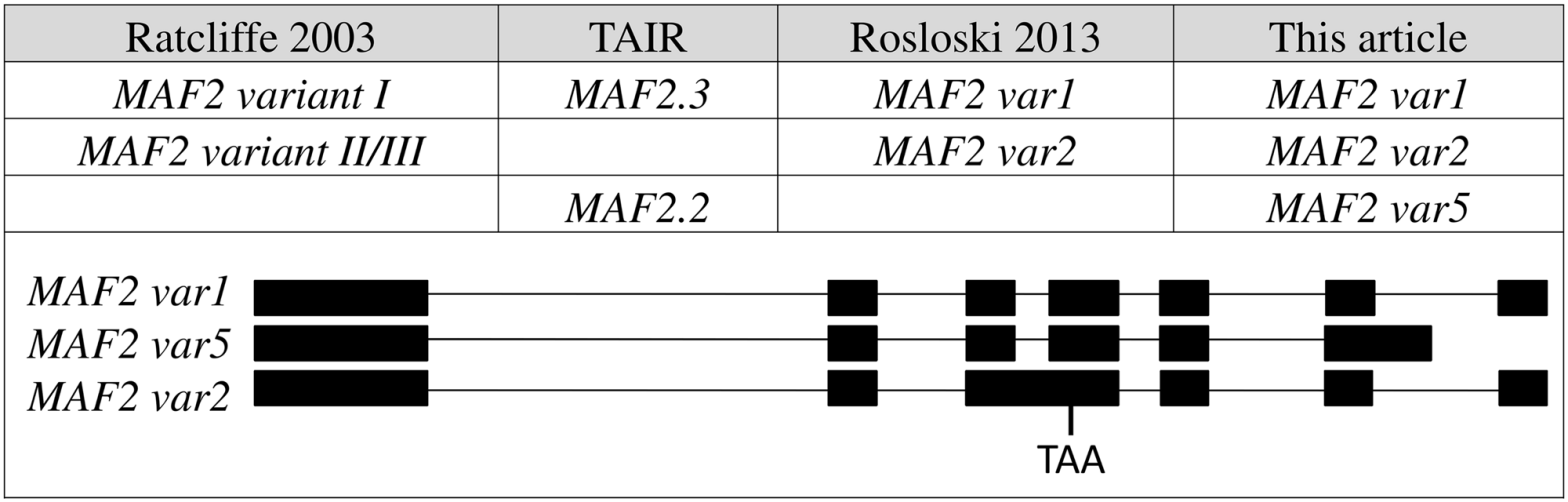
*MAF2* splice variants. *MAF2* splice variants observed by RT-PCR are shown with the names used in this report and alternative names reported elsewhere (Rosloski 2013, Ratcliffe 2003 and TAIR website). The names are followed by a schematic representation of the genomic structure of each splice variant. Rectangles represent exons and lines represent introns. The premature termination codon introduced as a result of intron 3 retention in *MAF2var2* is indicated (TAA).

Several alternatively spliced isoforms of *MAF2* have either been identified or predicted (Fig 2 and S2 Fig). Over-expression of one of these isoforms, *MAF2var1*, has previously been reported to delay flowering in the *Arabidopsis* L1-2 accession [30] (an accession that lacks a functional FLC [33]) in short days. Unexpectedly, overexpression of *MAF2var1* in *Col* resulted in early flowering, a result that was attributed to non-target effects of *MAF2var1* expression on *MAF* paralogs, leading to artifactual early flowering [30]. Co-suppression problems have therefore precluded *MAF2* splice variant function from being analysed in the *Col* background. We have repeated these experiments in *Col* (S3 Fig) and found that plants overexpressing *MAF2var1* do indeed flower later than wild type plants (Fig 1B), in agreement with *MAF2* acting as a repressor of flowering. However, over-expressing *MAF2var1* in an *svp* background does not result in repression of flowering, since 35S::*MAF2var1 svp* plants flower early and at the same time as *svp* mutants (Fig 1B). Taken together, these results indicate that MAF2, like its close relatives FLC and FLM, directly interacts with SVP to repress flowering. We also tested the extent to which MAF2 and FLC are responsible for the repression of flowering exerted by SVP at 21°C. *flc maf2* double mutants flower slightly later than *svp* mutants (Fig 1C), suggesting that SVP may still be capable of repressing flowering at 21°C in the absence of both FLC and MAF2. These results are in accordance with findings that other proteins of the FLC subfamily, such as FLM, can interact with SVP [12].

### The effect of *maf2* mutation on *FT* in *Col*

SVP directly represses the floral integrators, *FT* and *SOC1*, through binding to their promoters [11]. In previous experiments, over-expression of *MAF2* has also been shown to be capable of repressing both *SOC1* and *FT* [9][12]. In agreement with this, *FT* expression is elevated in *maf2* mutants (Fig 1D), although the increase in expression is small, reflecting the mild phenotype of the *maf2* mutant in the *Col* background (Fig 1A and 1D). Therefore, MAF2, like SVP, FLC and FLM is involved in the repression of *FT* [12].

### *MAF2* temperature-dependent alternative splicing

**Fig 3:**
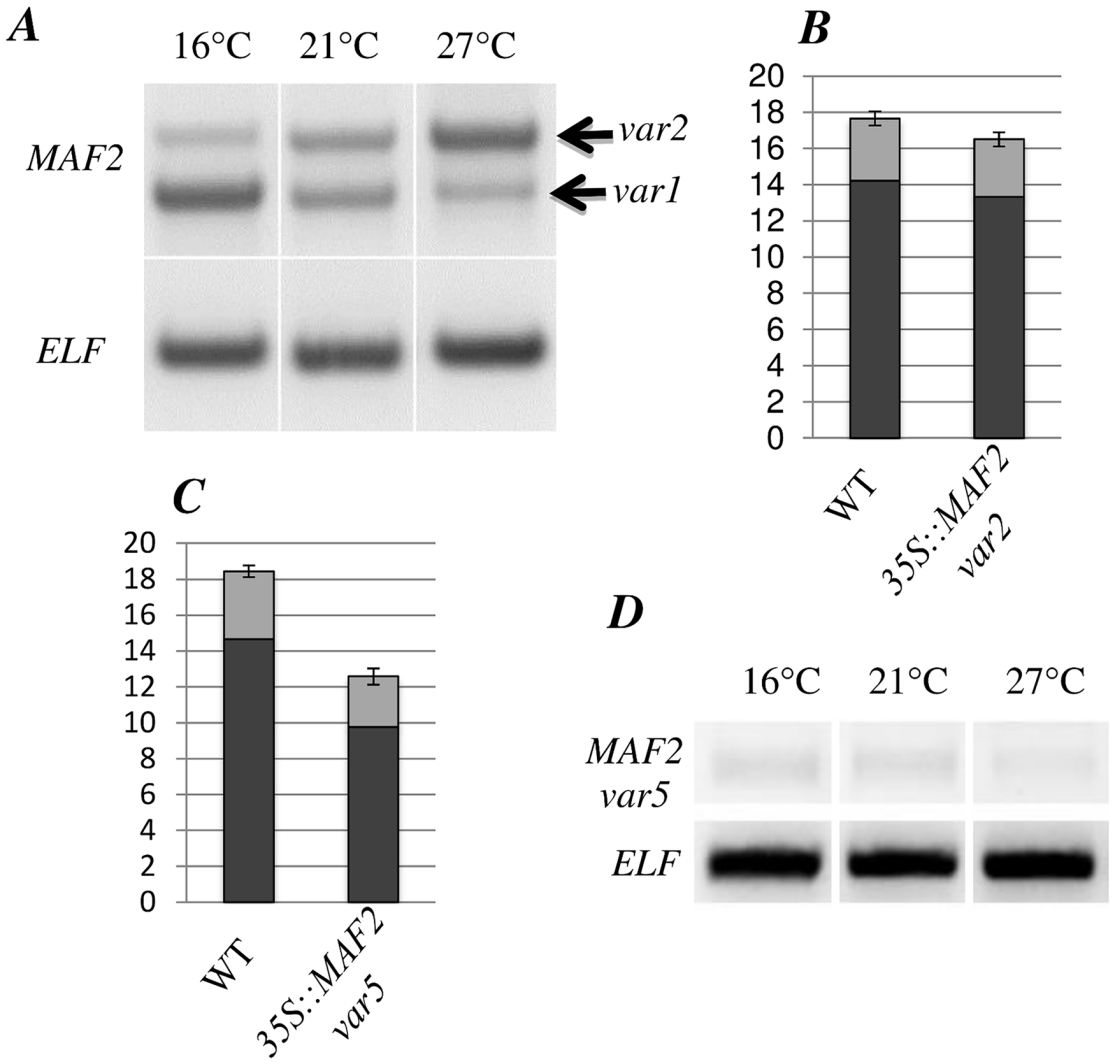
MAF2 splice variant analysis. A, D, Expression of *MAF2var1*, *MAF2var2* and *MAF2var5* splice variants at different temperatures analysed by RT-PCR. Elongation Factor (ELF) is used as a control. B, C, Flowering time of plants overexpressing *MAF2var2* or *MAF2var5* compared to WT at 21°C (no. of plants analyzed 47 and 53, four independent transformants for each transgenic line). The columns represent the number of rosette leaves (in black) plus the number of cauline leaves (in grey). Error bars represent the standard error.

The steady-state levels of the *MAF2* splice variants change in response to ambient temperature [14][30]. We analyzed the relative expression levels of previously published *MAF2* splice variants (Fig 2 and S2 Fig) at 16°C, 21°C and 27°C (Fig 3A). When grown at 16°C *MAF2var1*, the isoform that acts as a repressor of flowering in *Col*, predominates (Fig 3A), as was previously demonstrated for plants grown at 4°C [30]. Our analysis at 16°C shows that even a slight decrease in temperature results in this splice isoform predominating. As previously reported in Rosloski et al. 2012, the steady state levels of the splice variants *MAF2var1* and *MAF2var2* are approximately equal at 21°C. However, when plants are grown at 27°C a switch in splice preference is observed, so that *MAF2var2* now predominates at the expense of *MAF2var1*. Therefore, over a small physiological temperature range of just eleven degrees the dominant splice isoform switches from *MAF2var1* (at 16°C) to *MAF2var2* (at 27°C).

Since we showed that MAF2 requires SVP to repress flowering, we used yeast two-hybrid analysis to test the ability of *MAF2* splice variants to encode proteins capable of interacting with SVP. *MAF2* has been predicted to generate several distinct splice variants ([9], TAIR, S2 Fig). Despite extensive RT-PCR analysis under a range of conditions, we were only able to detect the 3 splice variants shown in Fig 2 in plants, but we artificially generated the undetectable MAF2var6 isoform for completeness. Testing splice variants in yeast two-hybrid assays revealed that MAF2var1, MAF2var5 and MAF2var6 are capable of interacting with full length SVP, but a MAF2var2-SVP interaction was not detected (S2 Fig). MAF2var2 lacks part of the K-domain and all of the C-domain; regions that are involved in the homo- and hetero-dimerisation of MADS-box transcription factors [34][35] and it is likely that exclusion of these domains from the *MAF2var2* isoform renders the resulting protein incapable of forming heterodimers.

### The *MAF2var2* and *MAFvar5* splice variants

*MAF2var2*, the isoform that predominates at high temperatures, was tested for its ability to repress flowering by over-expression. In agreement with Rosloski et al., 2013, *35S*::*MAF2var2 Col* plants flower at the same time as wild type controls, indicating that this form, unlike *MAF2var1*, is incapable of repressing flowering (Fig 3B). This finding is consistent with the yeast two-hybrid data that shows that MAF2var2 does not interact with SVP. The intron retention event (Fig 2) that produces *MAF2var2* also introduces a premature termination codon that could potentially trigger nonsense-mediated mRNA decay (NMD) of this splice variant, leading to degradation of the mRNA and suppression of its steady-state levels [36][37]. However, expression of *MAF2var2* does not significantly increase in the NMD mutants *upf1-5*, *upf3-1* and *smg7-1* (S4 Fig), indicating that *MAF2var2* is not targeted by NMD. Indeed, none of the *MAF2* splice variants appear to be degraded by NMD (S4 Fig). Therefore, although premature stop codons are recognised triggers of NMD in some transcripts and there are examples, such as *LHY-UAS4* [38], where temperature regulated splicing produces variants that trigger NMD, *MAF2var2* is not targeted by NMD under the conditions tested here. From these analyses we conclude that in wild type plants exposed to elevated temperatures TD-AS favours the production of *MAF2var2*, a non-functional splice isoform that serves only to decrease the levels of *MAF2var1*, which would otherwise actively repress flowering.

The third detectable *MAF2* splice variant, *MAF2var5*, which skips the sixth intron and introduces a premature termination codon that removes the C-terminal domain of the protein (Fig 2), was also assessed for its ability to control the floral transition. Unexpectedly, *35S*::*MAF2var5* plants flower earlier than wild type (Fig 3C), suggesting that *MAF2var5* can promote flowering. MAF2var5 is capable of interacting with SVP (S2 Fig) and could therefore promote flowering by competing with FLM for the available SVP, with the resulting complex unable to repress flowering, analogous to the proposed mode of action of the FLM-δ splice variant [27]. However, RT-PCR reveals very low expression of *MAF2var5* at all tested temperatures in *Col* (Fig 3D), calling its contribution to flowering time in this accession into question. Further analysis will be required to determine the significance of *MAF2var5*, if any, in regulating the floral transition and to assess if this variant plays a significant role in other accessions.

### MAF2 represses flowering at low temperatures

**Fig 4:**
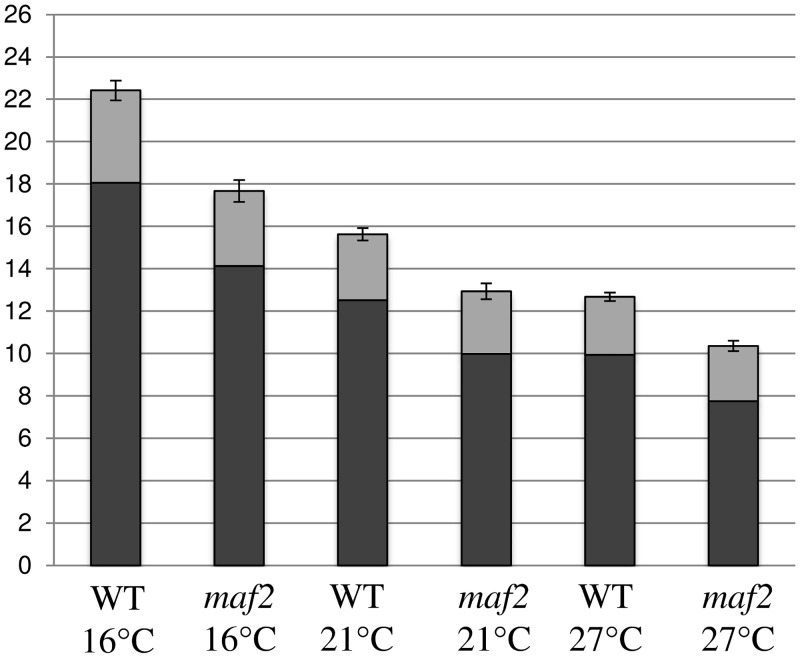
Flowering time of WT and *maf2* mutants at different temperatures. (no. of plants analyzed 257) The columns represent the number of rosette leaves (in black) plus the number of cauline leaves (in grey). Error bars represent the standard error.

MAF2var1 represses flowering and is the predominant isoform at lower temperatures. To study the contribution of MAF2 to the repression of flowering at different temperatures, *maf2* mutants were grown at 16°C, 21°C and 27°C and compared to wild type *Col* plants (Fig 4). *maf2* mutants grown at 16°C flower at around the same time as WT plants grown at 21°C and significantly earlier than WT plants grown at 16°C. Similarly, flowering of *maf2* mutants at 21°C is comparable to flowering times observed in WT plants at 27°C, indicating that the *maf2* mutant is deficient in its response to temperature change across a range of temperatures from 16°C to 27°C. *maf2* mutants retain a limited ability to respond to ambient temperature, since they flower later at 16°C than 27°C and this is likely to be due to the presence of *FLM* and *SVP*. Even at 27°C *MAF2* mildly represses flowering, as evidenced by *maf2* plants grown at 27°C flowering slightly earlier than WT plants grown under the same conditions (Fig 4). This residual repression is presumably caused by the limited production of *MAF2var1* at 27°C (Fig 3A).

### MAF2 works in parallel with FLM to repress flowering at low ambient temperatures

**Fig 5:**
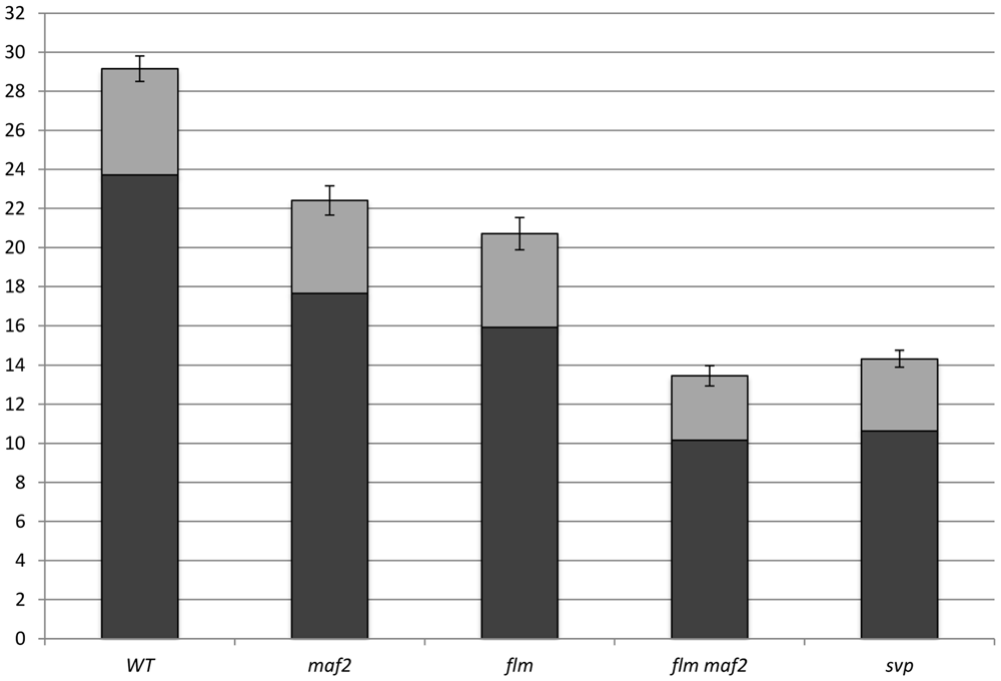
Flowering time of WT and *maf2*, *flm*, *flm maf2* and svp mutants at 16°C. (no. of plants analyzed 147) The columns represent the number of rosette leaves (in black) plus the number of cauline leaves (in grey). Error bars represent the standard error.

FLM and MAF2 encode related MADS-box transcription factors that repress flowering at low temperatures. The early flowering phenotype observed in *flm* mutants grown at 16°C is comparable with that seen in *maf2* mutants, but *maf2 flm* double mutants show an additive effect, producing a very severe early flowering phenotype at 16°C (Fig 5). This suggests that *flm maf2* double mutants are very strongly compromised in their ability to repress flowering in response to low temperatures. Since both FLM and MAF2 repress flowering by interacting with SVP ([27], S2 Fig) and *svp* mutants are unable to respond to low temperature, we attempted to determine whether all of the low temperature repressive activity of SVP could be attributed to MAF2-SVP and FLM-SVP complexes. *flm maf2* double mutants and *svp* single mutants grown at 16°C show an almost identical severe early flowering phenotype (Fig 5) suggesting that SVP relies mainly on interaction with FLM or MAF2 to repress flowering in response to low ambient temperatures.

## Discussion

### The role of MAF2 in repressing flowering

MAF2 represses flowering; a function that becomes inactivated either by extended exposure to cold or increased ambient temperature [9][39][this article]. In the vernalization response, when exposure to low temperature decreases the expression of *FLC*, *MAF2* remains expressed for longer and is responsible for the maintenance of a repressed state in brief cold periods [9]. In the ambient temperature response, FLM and MAF2 repress flowering at low temperature, with the active *FLM* and *MAF2* splice variants decreasing in response to increasing temperature. SVP has previously been shown to interact with both FLC and FLM to repress flowering [11][27]. Here we demonstrate that MAF2 also requires SVP to repress flowering. Like FLC and SVP, MAF2 represses both *FT* and *SOC1*, possibly by directly binding their promoters as part of a complex with SVP, as is the case for FLC-SVP [11]. In *Col*, where the *FLC* transcript abundance is lower than in active FRI ecotypes, FLC causes a mild repressive effect on flowering, similar to the repression exerted by MAF2 and a comparable early flowering phenotype is seen in both *flc* and *maf2* mutants (S5 Fig). The mechanistic basis for this temperature response is, however, not the same, since *MAF2* is not regulated by FRI [9]. Unlike *FLC*, expression of *MAF2* does not increase in *Col* FRI^+^ plants (S5 Fig). In ecotypes such as *Col*, where FRI is inactive, FLC plays a limited role in repression of flowering and it is possible that *MAF2* plays a more prominent role.

### TD-AS controls flowering time

In addition to its role in the vernalization response, MAF2 represses flowering at low ambient temperature. However, the mechanisms through which vernalization and ambient temperature control *MAF2* to affect flowering time are completely different. Vernalization affects expression of the *MAF2* gene, whereas temperature acts post-transcriptionally on *MAF2* splicing. *MAF2* is therefore an example of a gene that is controlled by an environmental variable acting through two different mechanisms to influence flowering time. Ambient temperature affects *MAF2* splicing through TD-AS, by influencing the relative abundance of two main splice isoforms; a full length *MAF2* (*MAF2var1*) that represses flowering and a truncated *MAF2* (*MAF2var2*) which is inactive. This differential splicing involves increased retention of intron 3 at elevated temperatures (Fig 2). Increased intron retention at 27°C results in the production of more inactive *MAF2var2*, at the expense of the active repressor of the floral transition, *MAF2var1* (Fig 3A). Our splice site analysis adds to the published data [39][14] by broadening the temperature range to 27°C and confirming that the progressive increase in *MAF2var2* expression continues at temperatures above 21°C. Plants show a progressive temperature-dependent flowering effect, flowering earlier at 27°C than at 21°C and earlier at 21°C than at 16°C. Decreased production of the active *MAF2var1* isoform as the temperature increases mediates the concomitant decrease in floral repression. The residual delay in flowering observed in *maf2* mutants at 16°C is largely due to the presence of functional FLM in these plants (Figs 4 and 5).

*FLM*, like *MAF2*, is also subject to TD-AS, but despite the high sequence similarity between these two genes, TD-AS in *MAF2* and *FLM* shows several differences. TD-AS affects different parts of the encoded MAF2 and FLM proteins, producing an inactive and a dominant-negative variant respectively [27][39]. TD-AS involves exon skipping in *FLM* and intron retention in *MAF2* and the *MAF2* isoform that predominates at elevated temperatures contains a premature termination codon [39][27]. Even the rare *MAF2* splice variant, *MAF2var5*, which our experiments suggest has the potential to act as a dominant negative, differs from the FLM-δ dominant negative splice isoform. Whereas *MAF2var5* skips the sixth intron and introduces a premature termination codon, FLM-δ skips exon 2 [27]. MAF2 and FLM, which encode two closely related protein partners of SVP, presumably originated through gene duplication and still retain high sequence similarity, making it more likely that they would share the same TD-AS regulatory mechanism. Surprisingly, these differences indicate that, on the contrary, TD-AS mechanisms of thermosensitivity evolved independently in these genes.

Although temperature regulated splicing is a factor in regulating the expression of genes controlling plant adaptation to changing temperatures [40], its mode of action remains unclear. Proteins involved in splice site selection, such as serine/arginine-rich (SR) proteins could be critical for TD-AS [41][42][43][44]. SR proteins have one or two RNA recognition motifs and determine splice site selection in a concentration dependent manner by forming spliceosome complexes [45]. The expression of SR protein encoding genes is modified in response to temperature change [14][41][43] and the SR gene transcripts are themselves subject to TD-AS [42][43][44]. Furthermore SR mutants have various defects that also include flowering time alterations [46] [47]. It remains to be seen whether temperature-dependent changes in SR protein levels drive TD-AS of developmental regulatory genes such as *MAF2* and *FLM*.

*FLM* and *MAF2* are not the only examples of temperature dependent splicing affecting flowering time. The circadian clock regulates several genes responsible for influencing flowering time, such as CONSTANS (CO), that acts to regulate the floral integrator *FT* [48][49][50]. Alternative splicing of some circadian clock genes such as *LATE ELONGATED HYPOCOTYL* (*LHY*), *CIRCADIAN CLOCK ASSOCIATED1* (*CCA1*), is regulated by temperature [38][51][52]. These TD-AS events can produce transcripts that are targeted by NMD, as in the case of *LHY* or generate protein forms that act as a dominant negative, such as *CCA1*. It will be interesting to discover the full range of TD-AS targets and the nature of the mechanism(s) that link ambient temperature to the choice of splice sites.

### FLM and MAF2 repress flowering in parallel at low temperature

The additive effect observed in the *flm maf2* double mutant (Fig 5) indicates that FLM and MAF2 work in parallel to repress flowering at 16°C. In addition, the fact that both FLM and MAF2 interact with SVP and the similarity of the early flowering phenotype seen at 16°C in *flm maf2* double mutants and *svp* single mutants, suggests that all the temperature responsiveness of SVP is mediated by its related interaction partners MAF2 and FLM. This agrees with previous findings [12] that show that the extremely weak responsiveness of the *svp* mutant to temperature change is similar to the responsiveness of *flm maf2 flc* mutants. It has been suggested that other members of the FLC clade can also weakly influence thermosensitivity [12], although currently *MAF2* and *FLM* are the only two that have been shown to be regulated by small changes in ambient temperature. This, together with our observation that the *flm maf2* double mutant flowers similarly to the *svp* mutant suggests that MAF2 and FLM are the two main FLC clade proteins acting in complex with SVP to determine sensitivity to ambient temperature. Further studies will be required to address the additional contributions of each member of the FLC clade and their mode of action in controlling thermosensitivity.

At 16°C both FLM-SVP and MAF2-SVP repress flowering. As the temperature increases FLM-mediated repression of flowering appears to lift before MAF2-mediated repression, since at 27°C *flm* mutants flower at approximately the same time as WT plants, whereas *maf2* mutants still flower slightly earlier ([14], S6 Fig). The parallel independent regulation of temperature induced flowering shown by *FLM* and *MAF2* is analogous to the independent parallel repression of flowering prior to vernalization caused by *FLC* and *MAF2*. In both cases *MAF2* adds an additional layer of repression to the control of the floral transition. Since MAF2 is capable of interacting with FLM in addition to its interaction with SVP [12] and MADS-box proteins have been found to be part of very large complexes [53], all three proteins could be part of a larger complex. If this is the case, depleting individual members of the complex could have differential effects, depending on their functional requirement within the complex. For example, the limited effect of individual *maf2* or *flm* mutations, compared to the more significant effect of mutating *svp*, could indicate a requirement for SVP in the complex, but a degree of redundancy between MAF2 and FLM in complex formation. Future experiments should focus on the composition of the complexes that repress flowering at low temperature and the changes induced when the abundance of components of the complex are changed in response to increases or decreases in temperature.

## Materials and Methods

### Yeast two-hybrid analyses

Yeast two-hybrid analysis was performed using the yeast strain PJ69-4 and *MAF2* splice variants were cloned in pDEST22 and pDEST32 [54]. Bait and prey plasmids were transformed into two different yeast mating types and mated to obtain the required combinations. Yeast was grown at 21°C on selective media to test for interactions (S2 Fig).

### Plant materials

*Arabidopsis Columbia* seeds (*Col*) were stratified on filter paper at 4°C for three days and then transferred to soil. Plants were grown in growth chambers set at different temperatures (16°C, 21°C, 27°C) in a long day photoperiod (16 hours light, 8 hours dark) and exposed to the same light intensity (230 μmol m^-2^ s^-1^). The statistical significance of differences in flowering time was verified using a two-tailed Student’s t-test.

### Genotyping

*maf2* mutants were provided by NASC (SALK_045623), the expression of *MAF2* was tested by RT-PCR and found to be undetectable in the *maf2* mutant.

The following primers were used for genotyping: *maf2* SALK_045623 CA245 Atp58, MAF2 WT allele CA245 CA272, s*vp-32* CA345 AtP58, SVP WT allele CA345 CA346, *flc-101* CA352 Atp58, FLC WT CA351 CA352, *flm-3* CA379 AtP58, FLM WT allele CA378 CA379. Primer sequences can be found in S1 Table.

### Real time PCR

Quantitative real time PCR was performed with a Bio-Rad CFX96 real-Time System. Retrotranscription was performed with Superscript II Invitrogen retrotranscriptase on total RNA extracted using RNeasy (Qiagen). Relative expression was calculated using the Bio-Rad CFX Manager 3.0 software. Three biological replicates were performed for each experiment. Primers used for Real time quantitative PCR: MAF2 CA279 CA280, SVP CA384 CA385, FT CA388 CA389, FLC CA365 CA366, ACTIN primers ACT2F ACT2R. The primer sequences can be found in S1 Table.

### Semi-quantitative PCR to identify splice variant expression

Semi-quantitative PCR was performed by running a sample of the PCR on an agarose gel at different cycle points, to verify that the PCR had not reached the plateau phase. Elongation factor was used for normalization. PCRs of *MAF2* were run on a 3% agarose gel to separate the splice variants. The primers used were: ELONGATION FACTOR ELF1A1 ELF1A2, MAF2 var1/var2 CA339 CA340, MAF2 var5 CA339 CA284. The primer sequences can be found in S1 Table. Leaves of young plants (7 leaves) were used for the RNA analyses. Experiments conducted on later leaves gave comparable results.

### Overexpression of *MAF2* splice variants

Overexpression of *MAF2* splice variants was achieved by subcloning the appropriate coding sequence into the alligator vector pFP101 containing a Gateway recombination site downstream of the constitutive 35S promoter [55]. Transgenic seeds were identified by observing the GFP fluorescence in the seeds, only seeds showing a strong signal were sown on soil.

## Supporting Information

S1 Fig Quantitative real time RT-PCR of *SVP* and *MAF2* in WT and mutant backgrounds. Expression is presented relative to WT.
(TIF)

S2 Fig *MAF2* splice variants and yeast two-hybrid results. A, Published names used for the *MAF2* splice variants in different sources: [9] [30]. B, Results of yeast two-hybrid analysis of protein-protein interactions. For completeness we also generated *MAF2var6* by assembly PCR, although we were unable to detect this variant in plants. “+” indicates growth of yeast on selective media lacking leucine, tryptophan and histidine, which is indicative of a protein-protein interaction. Representative yeast growth plates are presented on the right, showing both yeast viability (-L-W plates) and interaction tests (-L-W-H plates).
(TIFF)

S3 Fig RT-PCRs on WT and lines overexpressing *MAF2* splice variants (OV) in WT and *svp* backgrounds. Elongation Factor (*ELF*) is used as a control. All OV samples show a significant increase in expression of the *MAF2* splice isoform compared to the WT. Note that despite *MAF2var1 svp* showing higher levels of *MAF2var1* expression than WT, these plants flower early, like *svp* (Fig 1B).
(TIF)

S4 Fig RT-PCR of *MAF2* splice variants in WT and NMD mutant backgrounds. In the NMD mutants *upf1-5*, *upf3-1* and *smg7-1*, *MAF2* splice variants are not expressed at significantly higher levels than in the WT. Elongation Factor (*ELF*) is used as a control.
(TIF)

S5 Fig Comparison between *maf2* and *flc* mutants. A, Flowering time of WT, *maf2* and *flc* mutants. The plants were grown in LD at 21°C and were not exposed to vernalization (no. of plants analyzed 65). The bars represent the number of rosette leaves (in black) plus the number of cauline leaves (in grey). The error bars represent the standard error. B,C Quantitative real time RT-PCR of, B, *MAF2*, and, C, *FLC* in WT and FRI (*Arabidopsis Col* with an active FRI) background. The graph shows the relative expression compared to WT plants.
(TIF)

S6 Fig Flowering time of WT, *maf2* and *flm* mutants at 27°C. (no. of plants analyzed 93) The columns represent the number of rosette leaves (in black) plus the number of cauline leaves (in grey). The error bars represent the standard deviation.
(TIF)

S1 Table Primers used in PCR experiments. (XLSX)
