# Supplementary figures and images for: MAF2 Is Regulated by Temperature-Dependent Splicing and Represses Flowering at Low Temperatures in Parallel with FLM
Source: PLoS One. 2015 May 8;10(5):e0126516. doi: 10.1371/journal.pone.0126516 (PMC4425511; doi:10.1371/journal.pone.0126516)

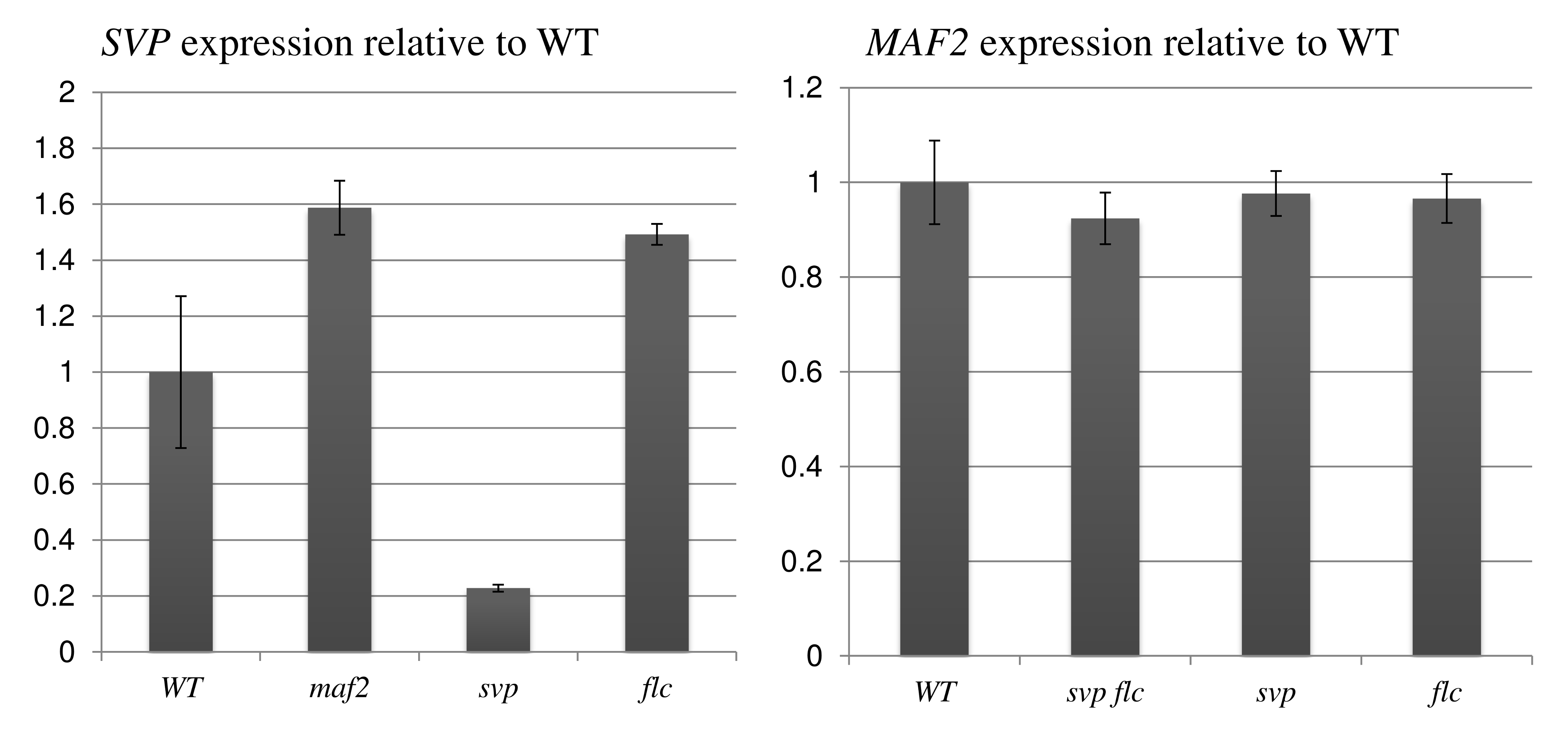

Supplement: S1 Fig — Expression is presented relative to WT. (TIF) [file pone.0126516.s001.tif]

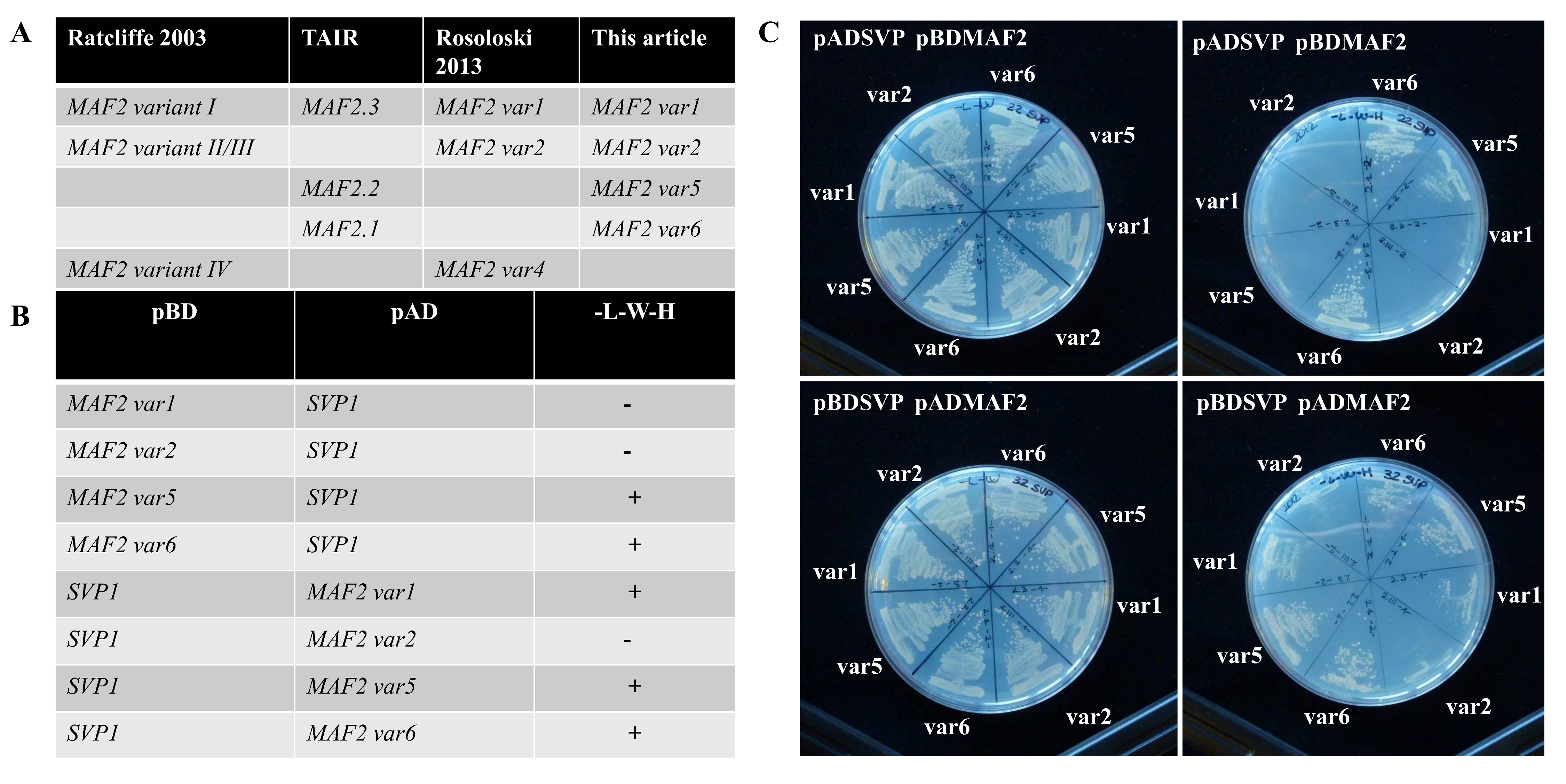

Supplement: S2 Fig — A, Published names used for the MAF2 splice variants in different sources: [9] [30]. B, Results of yeast two-hybrid analysis of protein-protein interactions. For completeness we also generated MAF2var6 by assembly PCR, although we were unable to detect this variant in plants. “+” indicates growth of yeast on selective media lacking leucine, tryptophan and histidine, which is indicative of a protein-protein interaction. Representative yeast growth plates are presented on the right, showing both yeast viability (-L-W plates) and interaction tests (-L-W-H plates). (TIFF) [file pone.0126516.s002.tiff]

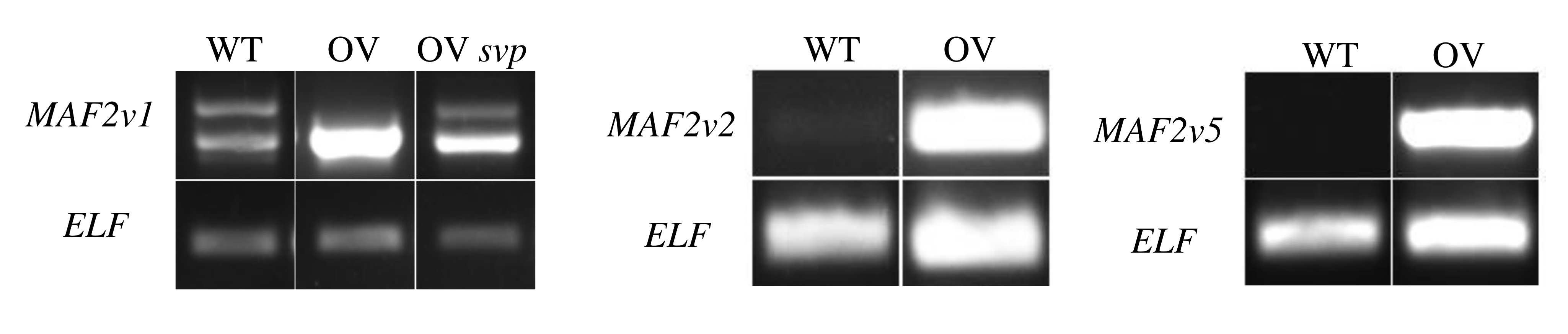

Supplement: S3 Fig — Elongation Factor (ELF) is used as a control. All OV samples show a significant increase in expression of the MAF2 splice isoform compared to the WT. Note that despite MAF2var1 svp showing higher levels of MAF2var1 expression than WT, these plants flower early, like svp (Fig 1B). (TIF) [file pone.0126516.s003.tif]

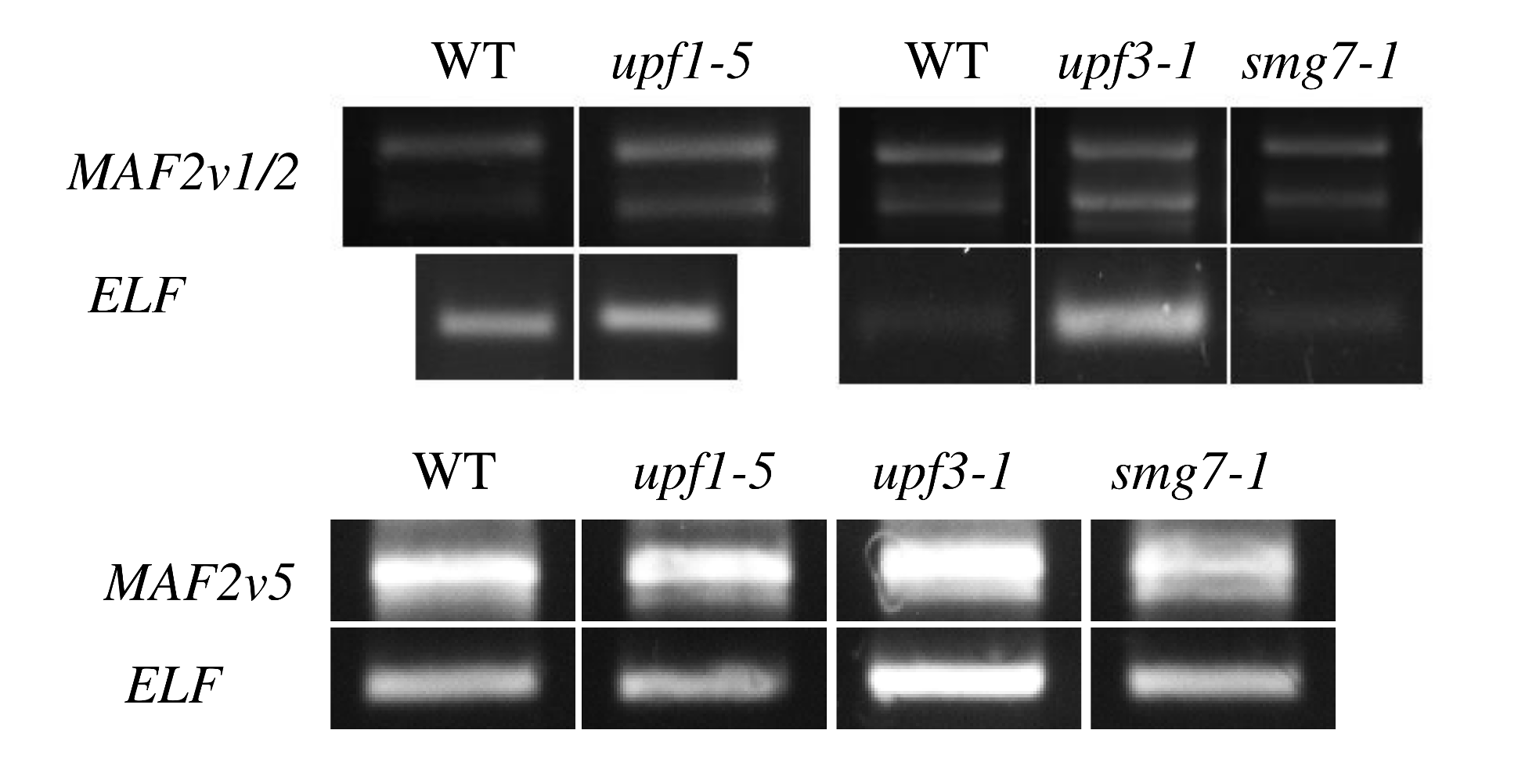

Supplement: S4 Fig — In the NMD mutants upf1-5, upf3-1 and smg7-1, MAF2 splice variants are not expressed at significantly higher levels than in the WT. Elongation Factor (ELF) is used as a control. (TIF) [file pone.0126516.s004.tif]

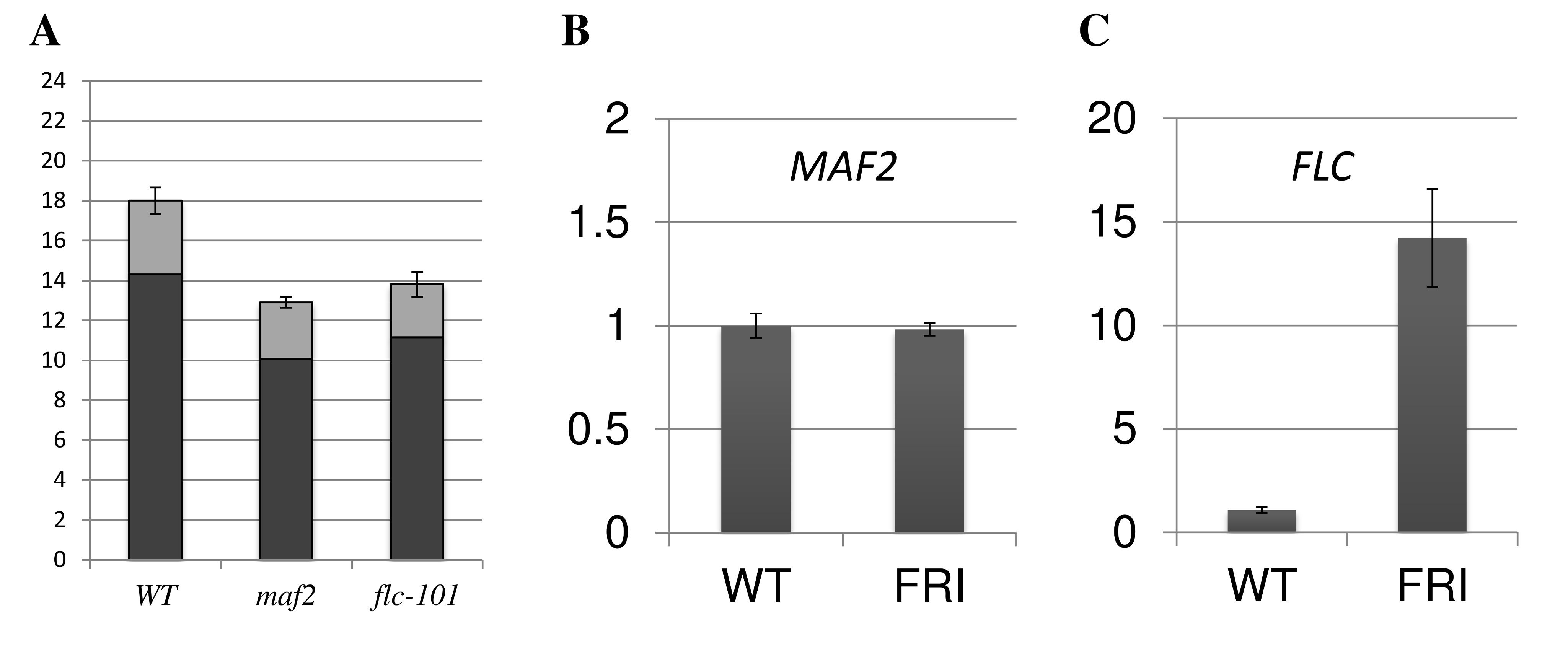

Supplement: S5 Fig — A, Flowering time of WT, maf2 and flc mutants. The plants were grown in LD at 21°C and were not exposed to vernalization (no. of plants analyzed 65). The bars represent the number of rosette leaves (in black) plus the number of cauline leaves (in grey). The error bars represent the standard error. B,C Quantitative real time RT-PCR of, B, MAF2, and, C, FLC in WT and FRI (Arabidopsis Col with an active FRI) background. The graph shows the relative expression compared to WT plants. (TIF) [file pone.0126516.s005.tif]

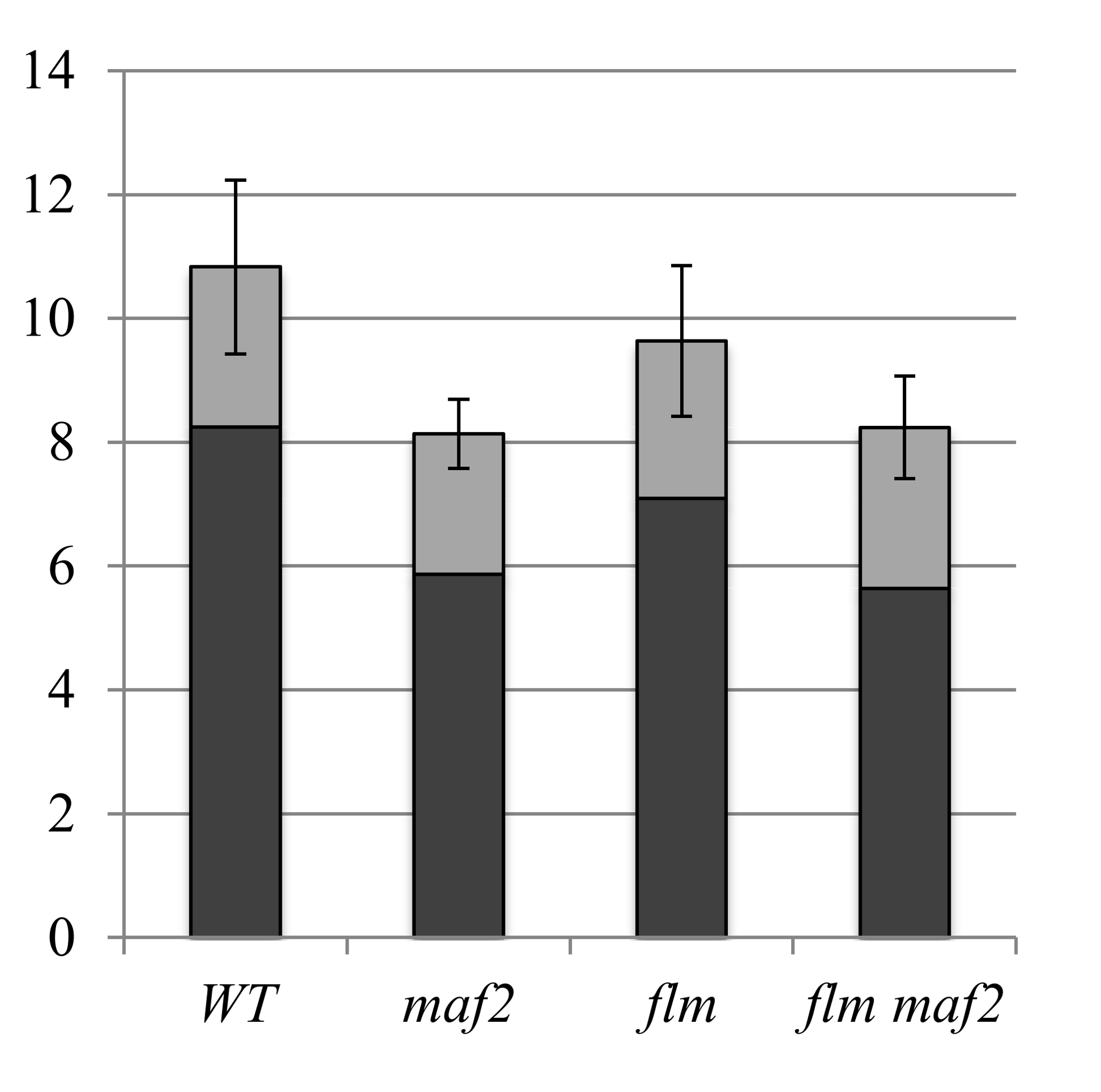

Supplement: S6 Fig — (no. of plants analyzed 93) The columns represent the number of rosette leaves (in black) plus the number of cauline leaves (in grey). The error bars represent the standard deviation. (TIF) [file pone.0126516.s006.tif]
